# Patterns and drivers of vector-borne microparasites in a classic metapopulation

**DOI:** 10.1017/S0031182023000677

**Published:** 2023-09

**Authors:** Laura S. Mackenzie, Xavier Lambin, Emma Bryce, Claire L. Davies, Richard Hassall, Ali A. M. Shati, Chris Sutherland, Sandra E. Telfer

**Affiliations:** School of Biological Sciences, University of Aberdeen, Aberdeen, UK

**Keywords:** classic metapopulation, connectivity, dispersal, distance-dependent, host, life-history, vector, vector-borne

## Abstract

Many organisms live in fragmented populations, which has profound consequences on the dynamics of associated parasites. Metapopulation theory offers a canonical framework for predicting the effects of fragmentation on spatiotemporal host–parasite dynamics. However, empirical studies of parasites in classical metapopulations remain rare, particularly for vector-borne parasites. Here, we quantify spatiotemporal patterns and possible drivers of infection probability for several ectoparasites (fleas, *Ixodes trianguliceps* and *Ixodes ricinus*) and vector-borne microparasites (*Babesia microti*, *Bartonella* spp., *Hepatozoon* spp.) in a classically functioning metapopulation of water vole hosts. Results suggest that the relative importance of vector or host dynamics on microparasite infection probabilities is related to parasite life-histories. *Bartonella*, a microparasite with a fast life-history, was positively associated with both host and vector abundances at several spatial and temporal scales. In contrast, *B. microti*, a tick-borne parasite with a slow life-history, was only associated with vector dynamics. Further, we provide evidence that life-history shaped parasite dynamics, including occupancy and colonization rates, in the metapopulation. Lastly, our findings were consistent with the hypothesis that landscape connectivity was determined by distance-based dispersal of the focal hosts. We provide essential empirical evidence that contributes to the development of a comprehensive theory of metapopulation processes of vector-borne parasites.

## Graphical abstract

**Figure:**
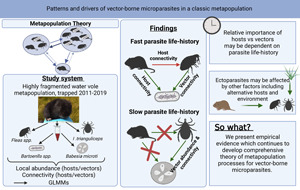

## Introduction

A fundamental prediction of disease ecology is that the prevalence and persistence of a parasite depend on the abundance or density of susceptible hosts and infectious agents in a population (Begon *et al*., 2002; Hagenaars *et al*., 2004; Lloyd-Smith *et al*., 2005; Telfer *et al*., 2007). In classic metapopulations, where local host subpopulation sizes are small, the probability of stochastic extinction of host subpopulations (Hanski, 1994), and simultaneous extinction of any associated parasites (Lloyd-Smith *et al*., 2005), increases. Further, parasites themselves are prone to stochastic extinctions when scarce, and the 2 stochastic extinction processes compound (Lei and Hanski, 1997), such that the persistence and spread of the hosts and parasites in local subpopulations can only be understood at larger spatial scales.

Critically, inter-patch dispersal drives the recolonization of patches and reinfection of local subpopulations (Hanski and Simberloff, 1997). For parasites that are dependent on hosts for dispersal, successful parasite dispersal is influenced by the timing and length of infection (Cross *et al*., 2005; Daversa *et al*., 2017); parasites with longer infection periods or host-attachment periods during seasons of high host dispersal activity should have higher dispersal rates (Cross *et al*., 2005; Daversa *et al*., 2017). Effective dispersal in metapopulations is often quantified by some measure of connectivity (e.g., Sutherland *et al*., 2014). Connectivity is determined by the abundance of possible dispersers, the distance between patches and a species' dispersal ability. Hosts and parasites with large dispersal ranges increase connectivity between patches and should have more homogenous distributions across the metapopulation. Those with a smaller dispersal range should have more aggregated distributions (North and Godfray, 2017). Where parasites are dependent on their hosts for movement, host dispersal ranges will determine parasite connectivity (Watts *et al*., 2018). Thus, host and parasite dynamics, infection probability and parasite prevalence are predicted to relate to connectivity between patches and subpopulations (Hanski and Simberloff, 1997).

Vector-borne microparasites are reliant on both vectors and hosts which adds further complexity. Within subpopulations, transmission rates depend on the contact rates between hosts and vectors. Between subpopulations, microparasite spread and subpopulation (re)colonization is contingent on the dispersal rates of infected vectors or the probability that an infected host encounters a subpopulation with susceptible hosts and vectors. Thus, the dynamics of vector-borne microparasites are expected to be nested within those of the host and vector (Parratt *et al*., 2016).

**Figure 1.**
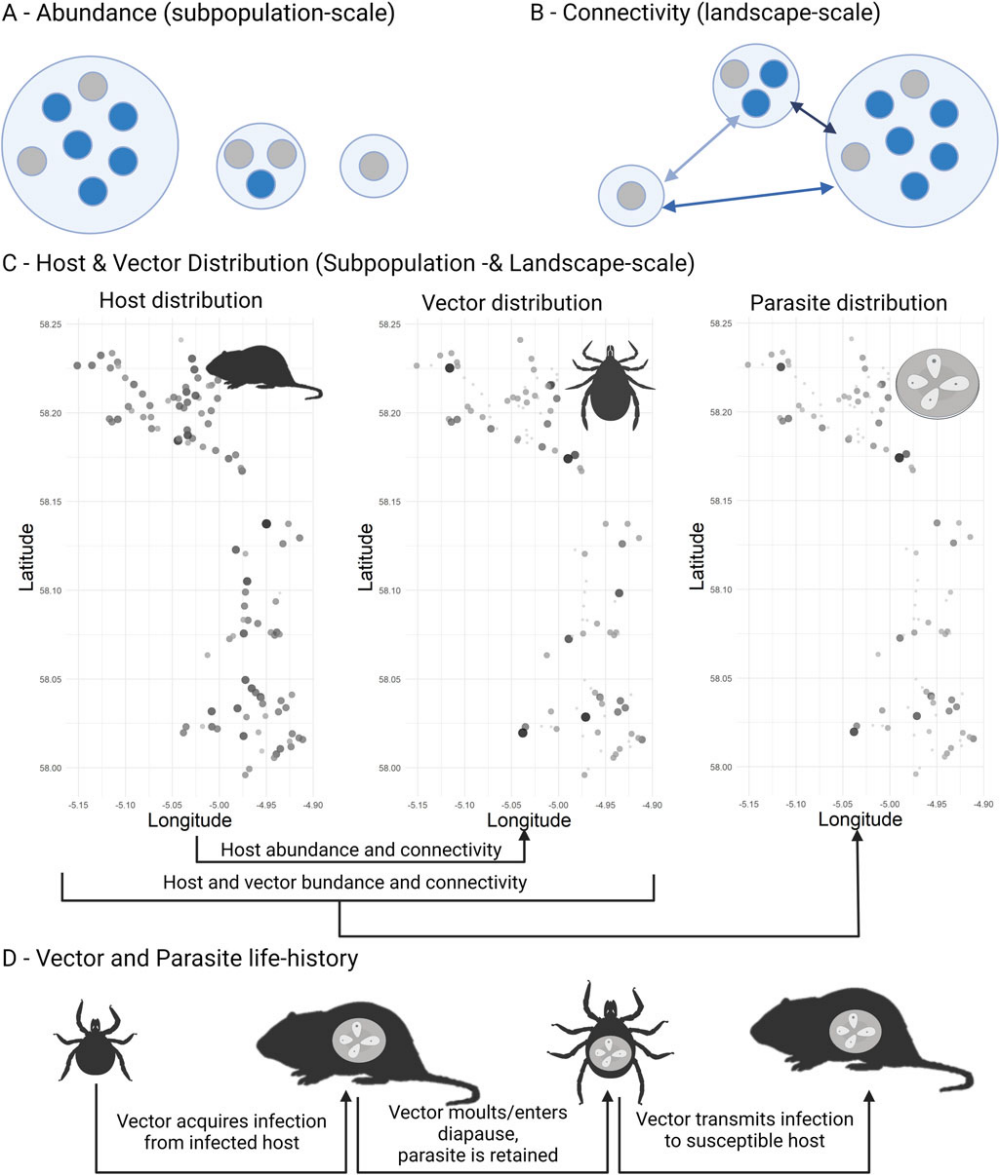
Conceptualization of drivers of infection dynamics in a metapopulation setting. Infected hosts are represented as blue circles (A, B). Locally, infection probability is driven by the abundance of suitable hosts (A). In a metapopulation setting, connectivity at the landscape-scale shapes infection spread and thus infection risk (B). Connectivity is shaped by the size of and distance between neighbouring subpopulations. When considering a vector-borne microparasite, both the abundance (represented by circle shading) and connectivity (represented by spatial position) of hosts and their associated vectors need to be taken into account (C, see also Fig. 4). Parasite transmission dynamics should therefore be nested within both host and vector dynamics. Note that host abundance and connectivity may shape vector distribution, especially if the vector is dependent on hosts for long-distance dispersal. Lastly, microparasite transmission is dependent on the successful and successive transmission between vectors and hosts (D). Thus, delays in the transmission cycle, due to vector lifecycles or the entering of diapauses between feeding events, may induce time-lags between host, vector and microparasite dynamics. Created with BioRender.com.

Vector and microparasite life-histories and vector-feeding habits can further affect spatiotemporal patterns of parasite dynamics. For directly transmitted parasites it has been highlighted that those with fast transmission between hosts should be able to colonize uninfected subpopulations more successfully than those with longer time-lags, potentially due to an environmental stage, between infection of the host and onward transmission (Manlove *et al*., 2022). Analogously, time-lags introduced by vector-feeding behaviour and life-history may determine the speed of transmission and transmission rate, and thus parasite spread. Fleas feed successively on different hosts as adults, allowing for rapid transmission of microparasites. In contrast, *Ixodes* ticks feed once per life stage on a single host, while moult and questing times between feeds can span several months (Randolph, 2004), delaying onward transmission (Fig. 1). Moreover, some ectoparasite vectors may experience diapause during their lifecycle, where host-seeking behaviours or development are suspended, allowing vectors to time their emergence to increase host encounters and survival (Gray *et al*., 2016). This can lead to a time-lag in infection dynamics of ectoparasites and associated vector-borne microparasites (Fig. 1, Telfer *et al*., 2007). Intriguingly, for parasites in classic metapopulations, diapause may further enable vectors and their microparasites to survive in the absence of their host, decoupling host and parasite dynamics (Gray *et al*., 2013). These diverse life-histories should be reflected in metapopulation processes, including colonization-extinction dynamics and parasites subpopulation- and metapopulation-scale infection rates (‘occupancy rates’, Manlove *et al*., 2022; van Dijk *et al*., 2022).

Considering the inherent complexities of vector-borne parasite dynamics, we lack empirical evidence of how host and vectors at different spatial and temporal scales affect infection patterns of vector-borne microparasites in fragmented populations. Due to their ubiquity and their importance as common emerging infectious diseases (Jones *et al*., 2008), a greater understanding of the spatiotemporal dynamics of vector-borne parasites in fragmented populations is needed (LaDeau *et al*., 2015). Here, we investigate spatiotemporal dynamics and drivers of 3 groups of ectoparasites (*Ixodes trianguliceps*, *Ixodes ricinus* ticks and a pooled group of ‘fleas’) and 3 vector-borne microparasites (*Babesia microti*, *Bartonella* spp. and *Hepatozoon*) in a classically functioning metapopulation of water voles (*Arvicola amphibius*) (Lambin *et al*., 2012). We consider patterns and drivers at 3 spatial scales (local: within a local population; landscape: connectivity between neighbouring populations within the dispersal distance of water voles; metapopulation: abundances across all populations) and temporal scales (current and lagged-by-1 year). For each parasite, we expected infected hosts to be aggregated locally and all, except for the host generalist *I. ricinus*, to be aggregated at a landscape-scale, consistent with water vole dispersal driving infection dynamics. We further expected parasites with fast life-histories (fleas and *Bartonella*) to have higher occupancy and colonization rates and prevalence within infected subpopulations than parasites with slow life-histories (ticks and *B. microti*).

For drivers of infection probability, we expected infection probabilities for all non-generalist parasites to increase with local water vole abundances and connectivity at the landscape-scale, and in the case of vector-borne microparasites to also increase with abundances of the appropriate vector at both scales. We expected parasite dynamics to reflect their life-histories; ticks and tick-borne infections are expected to be more strongly correlated with the abundance of infected hosts, and host and vector abundances in the previous year, than fleas and flea-borne microparasites. As very little is known about the life-history and traits of *Hepatozoon* spp. parasites, we did not make any predictions on the patterns of this microparasite.

## Materials and methods

The study system, located in Assynt, northwest Scotland (Lambin *et al*., 2012), covers approximately 140 km^2^ of mixed upland heath, blanket bogs and dry and wet grasslands, interlaced with a large waterway network. Suitable habitat for water voles – slow-flowing streams with grassy edges, burrowable soils and burrow entrances – encompasses 8% of waterways (Aars *et al*., 2001). These form 110 habitat patches (length = 90–3146 m), widely distributed across the study area, with a mean distance of 516 m to the nearest patch (range = 92–1711 m) and an average distance of 11 527 m between patches. The estimated effective dispersal distance of juvenile voles is 2.1 km (Sutherland *et al*., 2014).

Water vole habitat patches are used by other mammals, including red deer (*Cervus elaphus*), domestic sheep (*Ovis aries*) and field voles (*Microtus agrestis*). The latter is found in 10–70% of patches each year and on average accounts for 20% of small mammal captures within water vole habitat patches (median = 0%). As the extent of parasite transmission between the focal host and alternative hosts is unknown, and given water voles constitute the majority of rodent hosts, this study focuses on water voles as drivers of parasite infections.

Water voles are infected by several ecto- and microparasites. These diverse host–vector–parasite systems are ideal to explore some of the patterns and drivers, and to highlight potential differences between them. *Ixodes trianguliceps* is a rodent-associated tick, generally found in host burrows and nests (‘nidicolous’); however, there are some indications that larvae may be exophilic, questing for hosts outside the burrow entrance (Estrada-Peña *et al*., 2017). *Ixodes ricinus*, a host generalist, is known to parasitize a wider range of hosts, including small mammals, birds, lizards and larger mammals, including deer and sheep. Both tick species feed once per life stage, followed by long periods of off-host moulting and questing (Randolph, 2004). Both species can undergo behavioural and developmental diapause in the UK (Randolph, 1975; Gray *et al*., 2016). Due to this, their life cycle can last anywhere from 2 to 3 years for *I. ricinus*, or 2 to 5 years for *I. trianguliceps* (Randolph, 1995, 2004).

*Ixodes trianguliceps* is considered the main vector of *B. microti* (*sensu lato*) (Bown *et al*., 2008), an apicomplexan haemoparasite infecting rodents (Table S1.2). While infection in the tick vector only lasts 1 moult (i.e. only 1 transmission opportunity) (Gray *et al*., 2002), infection in the mammalian host is chronic. However, some laboratory studies suggest that transmission to the vector is only possible during the acute phase, lasting several days (Randolph, 1995; Gray *et al*., 2002), potentially lowering transmission rates from host to vector. However, the infectious period in wild rodents has not been determined.

Fleas parasitize a wide range of small-to-medium-sized burrowing mammals; however, most flea species are associated with 1 or a few principal hosts (Krasnov *et al*., 2003, 2004*a*). Rodent-associated fleas have fast life-histories, spanning a couple of weeks to months, depending on the host, environmental conditions and flea species (Krasnov, 2008). Further, adult fleas feed successively on several hosts. Fleas mainly encounter new hosts in nests or burrows but can be directly transmitted between individuals during physical encounters (Krasnov and Khokhlova, 2001). Some flea species undergo reproductive diapause as adults (Krasnov, 2008), while others diapause during the egg or pupal stages (Osácar *et al*., 2001), introducing seasonality into flea dynamics. Of the fleas parasitizing voles in the metapopulation, *Peromyscopsylla silvatica* is known to diapause (Vashchenok, 2014), whereas it is unclear whether the other flea species diapause.

Fleas are the vectors and potential reservoirs of rodent-associated *Bartonella* spp., a group of facultative intracellular, Gram-negative bacteria, which infect erythrocytes and endothelial cells in mammalian hosts (Gutiérrez *et al*., 2015). In susceptible hosts, bacteraemia lasts from a few weeks to several months during which infections can be transmitted, and there has been evidence of recrudescence in some wild rodents.

Little is known about *Hepatozoon* as a parasite (Table S1.2), including its main invertebrate and vertebrate hosts. *Hepatozoon*, an apicomplexan intraerythrocytic parasite, infects a wide range of vertebrate hosts including reptiles (Tome *et al*., 2013), birds and mammals (Smith, 1996). Their definitive hosts are blood-sucking arthropods. Infection of the intermediate vertebrate host occurs through the ingestion of infected arthropods, or, in the case of predators, of infected prey (Smith, 1996).

From 2011 to 2019, habitat patches were surveyed yearly from early July to mid-August for signs of water vole occupancy. Patches with fresh latrines (fecal deposits used to mark territories) were classified as occupied. Where possible, we conducted live trapping for 2–4 consecutive nights in occupied patches. We set a minimum of 3 baited Elliot traps (dimensions: 33 × 10 × 9 cm^3^), 20 m apart, close to occupancy signs. We checked traps within 24 h. Sutherland *et al*. (2012) suggest that a resident vole has a 0.95 probability of being trapped over 4 nights. Thus, the number of voles trapped was assumed to represent the local subpopulation size.

Voles were weighed and sexed. We removed any accessible ticks, avoiding the area around the eyes, and collected fleas by brushing voles with a pet comb over a water bath for up to 1 min. Ectoparasites were preserved in 95% ethanol. Given that ticks and fleas spend considerable time off-host (Krasnov *et al*., 2004*b*), this sampling represents a subset of the ectoparasites, leading to an underestimation of parasite prevalence.

We collected a 20–60 *μ*L blood sample from the tail tip of an opportunistic subset of voles, aiming to maximize the proportion of patches sampled. The collection of blood samples was conducted under a UK Home Office licence (Project License PP2865679). In total, we collected blood samples from 58% of water voles caught (33–85% per year) and 83% (68–95% per year) of trapped populations. Blood sera and red blood cell (RBC) pellets were separated by centrifugation at 20 000 rpm for 20 min and stored at −20°C.

### Parasite identification

Ticks were identified to the species level and life stage (larvae, nymph, adult) following Hillyard (1996). We identified fleas to the species level following Smit (1957). Four flea species were identified, namely *Megabothris walkeri*, *Peromyscopsylla silvatica spectabilis*, *Ctenophthalmus nobilis* and *Hystrichopsyllidae talpae talpae*. However, *M. walkeri* made up around 75% of identified fleas (Davies, 2014). To maximize our statistical power, and assuming common drivers, we analysed fleas as a single group.

To detect microparasite infections, RBCs were rehydrated using up to 20 *μ*L of distilled water. DNA was extracted from 10 *μ*L rehydrated RBC following Mackey *et al*. (1998), with the modification that we used 100 *μ*L of isopropanol to precipitate the DNA.

*B. microti* was detected using a nested polymerase chain reaction (PCR) described by Simpson *et al*. (2005), targeting an apicomplexan-specific 18S rRNA fragment. The full protocol is outlined in Supplementary materials S2. We visualized DNA fragments on a 3% agarose gel run at 145 V for 1 h. PCR products of approximately 650 base pairs (bp) and 700 bp were detected. The expected length of *Hepatozoon* DNA fragments for this assay is 700 bp (Simpson *et al*., 2005). We sequenced a subset of PCR products with different fragment lengths (Eurofins™) and compared the resulting sequences with submissions on the NCBI platform. Larger fragments consistently had a 99% sequence overlap with an unknown *Hepatozoon* species found in a snake (accession number gi|480311142|KC696568.1), while fragments of 650 bp length had a 100% sequence overlap with *B. microti* infecting small mammals in the Omsk region, Russia (accession number gi|1032563630|KU955532.1). Thus, we scored bands around 650 bp as positive for *B. microti* and bands of 700 bp length as positive for *Hepatozoon* infection. We did not find any mixed infections.

In samples from 2012 to 2019, we identified *Bartonella* infection using a genus-specific quantitative polymerase chain reaction, targeting a fragment of the *rnp*-gene. Details can be found in Supplementary Materials S2.

### Statistical analysis

For each ectoparasite group (*I. trianguliceps* of any life stage, *I. ricinus* of any life stage, fleas), the response variable was a binary indicator (1/0) for whether a vole had the ectoparasite collected. For microparasites (*B. microti*, *Hepatozoon* and *Bartonella* spp.), the response variable was a binary indicator (1/0) which signified whether a vole tested positive or negative for the microparasite.

For each parasite, we fitted generalized linear mixed models (GLMMs) with a logit link and binomial errors in the lme4 package (Bates *et al*., 2015). All models included 3 random effects: to capture variation in infection probability explained by environmental features in the patch (e.g. altitude, habitat), we included ‘PatchID’; to account for non-independence between animals caught in the same year, we also specified ‘Year’ as a random effect; to account for non-independence resulting from a patch in the same year, we additionally included ‘SubpopulationID’ as a random effect.

#### Broad spatiotemporal patterns of infection probability (analysis I)

Initially, to understand broad spatiotemporal patterns in parasite infection probabilities, we examined variance components (analysis I, ‘Spatiotemporal variance analysis’). Fitting a null model including intercept-only random effects, we compared how the variance in infection probabilities was partitioned between years, patches or subpopulations.

#### Spatiotemporal patterns of infection probability (analyses II and III)

Host attributes such as sex and age may be important determinants of the infection probability of the individual (Krasnov *et al*., 2005). Thus, in the first step, we considered the influence of vole weight, as a proxy for age, polynomial weight, as a non-linear age relationship, sex and any interaction between weight and sex. Individual-scale variables which best describe infection probability best were retained in all the following models (Table 1).

**Table 1.** Details of analysis performed on parasite data

Analysis	Response	Years	*N*_voles_	Fixed effects	Random effects
*Spatiotemporal variance analysis (analysis I): broad spatiotemporal patterns*					
Contemporary	Fleas	2011–2019	1830	None	Year, patch, subpopulation
	*Ixodes trianguliceps*	2011–2019	1830		
	*Ixodes ricinus*	2011–2019	1830		
	*Bartonella*	2012–2019	873		
	*Babesia microti*	2011–2019	1065		
	*Hepatozoon*	2011–2019	1065		
*Generalized linear mixed models: scale-associated contemporary infection patterns (analysis II)*					
Contemporary	Fleas	2011–2019	1830	Stage 1: individual-scale variables+ Stage 2: subpopulation-scale infection abundance landscape-scale infection connectivity metapopulation-scale infection abundance	Year, patch, subpopulation
	*I. trianguliceps*	2011–2019	1830		
	*I. ricinus*	2011–2019	1830		
	*Bartonella*	2012–2019	873		
	*B. microti*	2011–2019	1065		
	*Hepatozoon*	2011–2019	1065		
*Generalized linear mixed models: scale-associated lagged infection patterns (analysis III)*					
Lagged	Fleas	2012–2019	1266	Stage 3: competitive model from contemporary analysis+ subpopulation-scale infection abundance previous year landscape-scale infection connectivity previous year metapopulation-scale infection abundance previous year	Year, patch, subpopulation
	*I. trianguliceps*	2012–2019	1266		
	*I. ricinus*	2012–2019	1266		
	*Bartonella*	2013–2019	499		
	*B. microti*	2012–2019	635		
	*Hepatozoon*	2012–2019	635		
*Generalized linear mixed models: contemporary host and vector-centred analysis (analysis IV)*					
Contemporary	Fleas	2011–2019	1830	Stage 1: individual-scale+ Stage 2: host abundance subpopulation-scale host connectivity landscape-scale host abundance metapopulation-scale	Year, patch, subpopulation
	*I. trianguliceps*	2011–2019	1830		
	*I. ricinus*	2011–2019	1830		
	*Bartonella*	2012–2019	873	Stage 1: individual-scale+ Stage 2: host abundance subpopulation-scale host connectivity landscape-scale host abundance metapopulation-scale vector abundance subpopulation-scale vector connectivity landscape-scale vector abundance metapopulation-scale	Year, patch, subpopulation
	*B. microti*	2011–2019	1065		
	*Hepatozoon*	2011–2019	1065		
*Generalized linear mixed models: lagged host and vector-centred analysis (analysis V)*					
Lagged	Fleas	2012–2019	1266	Stage 3: competitive model contemporary analysis+ host abundance subpopulation-scale previous year host connectivity landscape-scale previous year host abundance metapopulation scale previous year	Year, patch, subpopulation
	*I. trianguliceps*	2012–2019	1266		
	*I. ricinus*	2012–2019	1266		
	*Bartonella*	2013–2019	715	Stage 3: best model contemporary analysis+ host abundance subpopulation-scale previous year host connectivity landscape-scale previous year host abundance metapopulation-scale previous year vector abundance subpopulation-scale previous year vector connectivity landscape-scale previous year vector abundance metapopulation-scale previous year	Year, patch, subpopulation
	*B. microti*	2012–2019	710		
	*Hepatozoon*	2012–2019	710		

To investigate spatiotemporal patterns, we considered if infection probability was associated with the abundance of voles infected with the focal parasite in the subpopulation (local-scale), connectivity to infected voles in neighbouring patches (landscape-scale) or total abundance of infected voles in the metapopulation (metapopulation-scale) in (i) the current year (analysis II, ‘Scale-associated contemporary infection patterns’) and (ii) both the current and previous year (analysis III, ‘Scale-associated lagged infection patterns’). Details of the analyses are outlined in Table 1.

#### Hosts and vectors as potential drivers of infection probability (analysis IV & V)

Individual-scale variables which best describe infection probability were included in these models (Table 1). We explored whether infection probabilities were driven by the abundance of hosts (water voles) and/or the abundance of relevant vectors (for microparasites only) at the local-, landscape- and metapopulation-scales in (i) the current (analysis IV, ‘Contemporary host and vector-centred analysis’) and (ii) both the current and the previous years (analysis V, ‘Lagged host and vector-centred analysis’). For microparasite infections with more than 1 potential vector (*B. microti* and *Hepatozoon* spp.), relevant vectors were considered both separately and together.

#### Model building and selection

In each analysis, a series of models including relevant additive combinations of covariates was built. All covariates were centred and scaled.

To evaluate the extent to which parasite dynamics were mediated by host dispersal distance, we contrasted landscape-scale connectivity measures with the metapopulation-scale total abundance. Landscape-scale connectivity weights the importance of neighbouring patches by distance, to reflect whether patches can easily be reached by dispersing hosts and parasites. In contrast, metapopulation-scale total abundance considered the scales of effects to be independent of distance. Therefore, connectivity and metapopulation measures from the same year were not included in the same models. Where vole dispersal is a strong determinant of spatiotemporal patterns, landscape-scale connectivity measures should provide a better model fit. In contrast, metapopulation-scale abundances should be more informative where temporal effects dominate.

Model selection was restricted to models that did not include combinations of variables that lead to high multicollinearity [variance inflation factor (VIF) > 3] (Zuur *et al*., 2010). Models in each step were ranked using Akaike information criterion adjusted for small sample size (AICc). In the next stage, the variables from the best model (lowest AICc) and any competitive models (as per the conditions below) were retained separately. To avoid interpreting overly complex models and potentially uninformative parameters, models were only considered competitive if they fulfilled the following 4 conditions (adapted from Leroux, 2019):

1. are within 2 delta AICc of the best model,
2. do *not* have 1 or more additional parameters than the best model, or any higher-ranked model,
3. do *not* only include a subset of parameters found in the best model and
4. do *not* only include a subset of parameters found in a model already considered competitive given criteria 1–3.

In the final analysis, parameters were estimated across the best and any competitive models using conditional model averages.

To assess variance explained by competitive models, we calculated theoretical marginal and conditional *R*^2^ values. Marginal *R*^2^ is the variance explained by fixed effects only, while the conditional *R*^2^ also takes the variance explained by random effects into account. We used the MuMIn package (v.1.46.0; Barton, 2022) for model selection, model averaging and calculation of variance explained. We used R version 4.1.1 for all statistical analyses (R Core Team, 2021).

#### Datasets

As not all subpopulations were trapped or sampled yearly, we considered reduced datasets for lagged analyses. For the analysis of scale-associated lagged infection patterns (analysis III), we excluded individuals from patches that did not have blood samples collected in the previous year, while for the lagged host and vector-centred analysis (analysis V) animals from patches that were occupied but not trapped in the previous year were excluded. Only models fitted to the same dataset (i.e. within each analysis) were compared using AICc.

#### Explanatory variables

*Local-scale.* To understand whether infections were aggregated locally and whether infection probability correlated with the local abundance of infected individuals in the previous year (analysis II–III), we considered 2 explanatory variables: the number of other voles in the local subpopulation (*i*) infected with the focal parasite in the current year (*t*) denoted 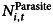 and the number of infected voles in the previous year (*t* − 1) denoted 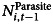.

As not all animals had a blood sample collected, we calculated the local abundance of a focal microparasite as follows:
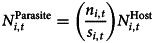
where *n*_*i,t*_ is the number of animals in subpopulation *i* that tested positive for a microparasite in the current year *t*, *s*_*i,t*_ is the number of animals sampled and 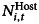 is the total number of voles caught in the local subopulation. When considering contemporary local abundances of infections, the focal individual was excluded.

To understand how local host and vector abundance within a local subpopulation may influence infection probability (analysis IV and V), we calculated the number of hosts caught 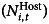 and the number of hosts infected with an ectoparasite vector 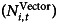 as a proxy. Due to the skewed distribution of ectoparasites on hosts, 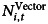 should be a good approximation for the contact rate with vectors in the local population. As there is no evidence that larvae can transmit *B. microti,* contemporary tick vector abundances only included nymphs and/or adults of each species. When considering the lagged effect of tick vectors, larvae were included. For patches unoccupied by the host in the previous year, lagged local-scale abundances 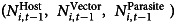 were 0, i.e., we assumed hosts, microparasites and vectors to have become extinct in the patch.

*Landscape-scale.* To test the hypothesis that the dispersal of hosts and parasites between neighbouring subpopulations shapes infection probabilities, we used a connectivity variable based on Hanski's connectivity metric (Moilanen and Nieminen, 2002). This estimates the distance-dependent influence of all populations on the focal subpopulation, by considering the abundance of the parameter of interest in neighbouring subpopulations 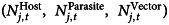 and estimating dispersal probabilities *via* a negative exponential dispersal kernel:
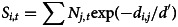
Here, *N_j,t_* was weighted by the neighbouring patch's (*j*) Euclidean distance, *d_i,j_*, from the centre of the focal patch (*i*). We used a fixed dispersal distance (*d*′) of 2.1 km, reflecting the previously estimated average dispersal distance of water voles in this system (Aars *et al*., 2006; Sutherland *et al*., 2014).

To calculate vector connectivity, we used the number of trapped hosts infected with the focal ectoparasites as an appropriate representation of between subpopulations mixing of ectoparasites, given that vectors rely on their hosts for dispersal.

*Metapopulation-scale.* Metapopulation-scale variables were calculated as the total abundance of the focal parameter in the metapopulation each year:
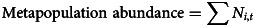


This corresponds to a scenario where temporal rather than spatial effects predict infection probabilities.

### Colonization/extinction rates

To quantify metapopulation turnover, we calculated water vole and parasite extinction and colonization rates. For water voles, we determined changes in the occupancy status of each patch between consecutive years based on latrine surveys. For each parasite, we determined changes in apparent occupancy where data on parasites were available for 2 consecutive years. Note that due to the multi-level sampling process, detectability was below 100%. Thus, apparent occupancy was certainly lower than true occupancy. Any patches unoccupied by the host were assumed to be unoccupied by the focal parasite. Patches occupied in 2 consecutive years (*t* and *t* + 1) ‘remained occupied’. A patch was ‘colonized’ if it was unoccupied in year *t* but occupied in year *t* + 1. Patches unoccupied in years *t* and *t* + 1 ‘remained unoccupied’. Lastly, if the patch was occupied in year *t* but unoccupied in *t* + 1 the focal parasite or host were deemed to have become ‘extinct’.

Colonization and extinction rates were then calculated as follows:
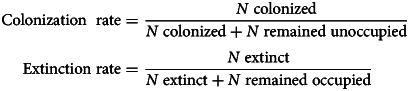
where *N* is the number of patches in each category each year (annual rate) or across all years (total rates).

We calculated the parasite colonization rate considering only newly colonized subpopulations, where the host was absent from the patch in the previous year. We compared this to the expected colonization rate under the assumption that each new subpopulation would have 2 founders:
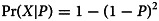
where *P* is the parasite prevalence each year and Pr(*X*|*P*) is the probability that at least one of the founders is infected with the parasite (see Supplementary materials S2.3 for a worked example).

## Results

**Figure 2.**
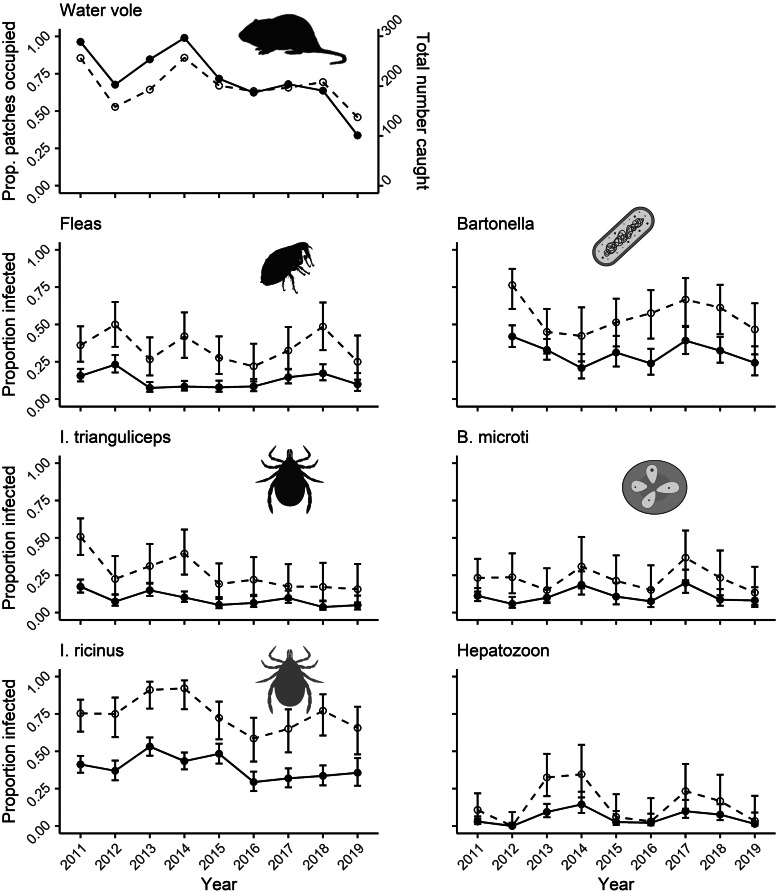
Top: Proportion of habitat patches found to be occupied by water vole hosts during latrine surveys (dashed line). The total number of voles caught (solid line) per year. Bottom: Proportion of voles (solid) and subpopulations (dashed) found to be occupied by each parasite. Created with BioRender.com.

We trapped 1941 water voles between 2011 and 2019. The number of voles caught per year ranged from 102 in 2019 to 298 in 2014. The proportion of patches occupied followed a similar pattern (Fig. 2). The median subpopulation size was 3 voles (range = 1–30), emphasizing the limited scope for parasite transmission within subpopulations. Host subpopulations experienced frequent turnover; the mean annual extinction rate of 0.25 (range = 0.05–0.44) was matched by a high colonization rate of 0.40 (0.20–0.75).

**Figure 3.**
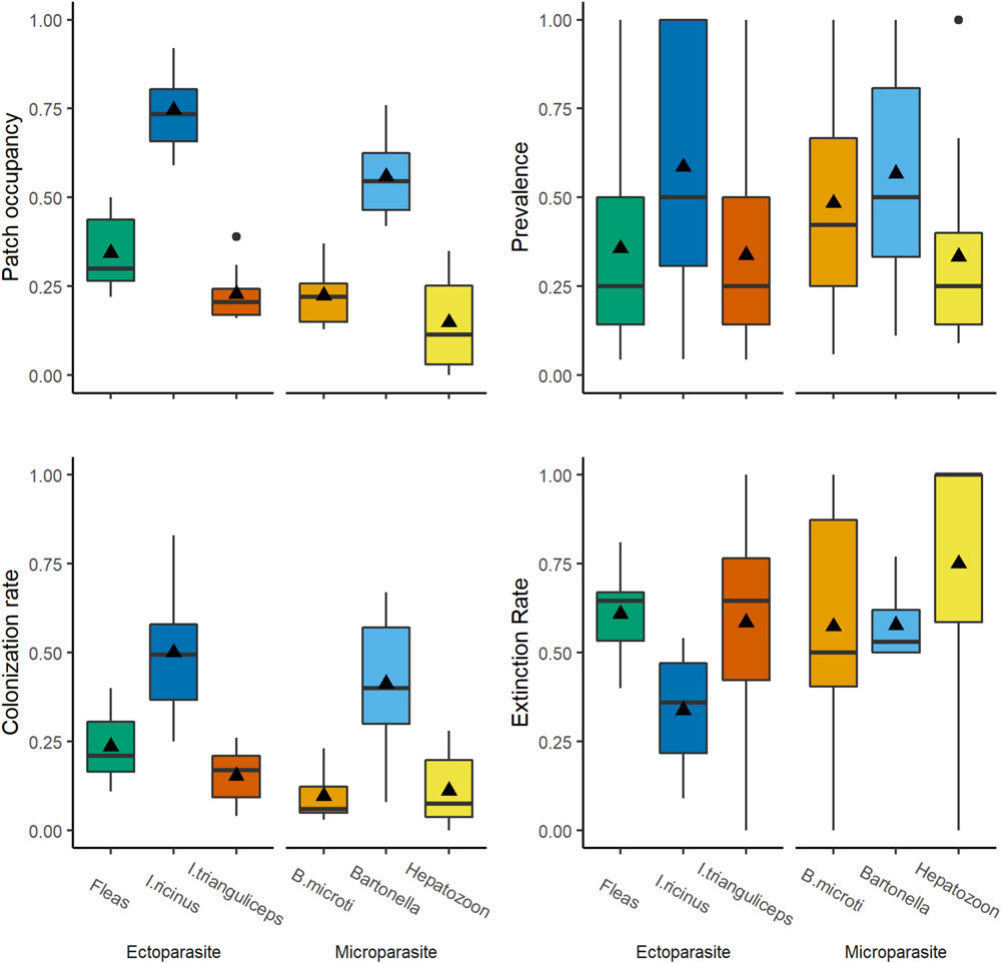
Metapopulation dynamics of fleas (green), *Ixodes trianguliceps* (orange), *Ixodes ricinus* (dark blue), *Bartonella* (light blue), *Babesia microti* (light orange) and *Hepatozoon* (yellow). Panels show the proportion of subpopulation occupied (top left), prevalence in infected subpopulations (top right), colonization rate (bottom left) and extinction rate (bottom right). Mean values are indicated by a triangle, median values are indicated by a horizontal line.

The host-generalist *I. ricinus* was the most prevalent ectoparasite parasitizing 40.3% of voles (total *n*-infected = 782) and 75% of subpopulations (*n* = 284), with an average occupancy rate of 0.75 (range = 0.59–0.92, Fig. 3). In contrast, rodent-specialist ectoparasites were less prevalent, with fleas parasitizing 12.5% (total *n*-infected = 242) and *I. trianguliceps* parasitizing 9.7%, (*n* = 188) of voles. Fleas infested 34% of subpopulations (*n* = 130), with an average occupancy rate of 0.35 (range = 0.22–0.50) and *I. trianguliceps* infested 28% of subpopulations (*n* = 105), with an average occupancy rate of 0.22 (range = 0.16–0.51, Fig. 3).

Similarly, within infested subpopulations, *I. ricinus* parasitized 59% of voles on average (range = 5–100%), with a median burden of 0 (0–25) per vole, compared to a prevalence of 36% for fleas (4–100%; median burden = 0, range = 0–8) and 34% for *I. trianguliceps* (4–100%; median burden = 0, range = 0–32) (Fig. 3). Overall, there was no clear difference between occupancy rates or prevalence of fleas and *I. trianguliceps* despite differing life-histories (Fig. 3A and B).

A total of 1094 voles were screened for microparasite infection from 2011 to 2019. Only blood samples collected from 2012 to 2019 were screened for *Bartonella* (n-screened = 899). *Bartonella* was the most prevalent microparasite infection (32.3%, *n*-infected = 290) contrasted to the less prevalent *B. microti* (10.8%, *n* = 118) and *Hepatozoon* (5.4%, *n* = 59) infections. *Bartonella* was further found in more than half of tested subpopulations (56%, *n* = 148), which was higher prevalence than *B. microti* (22%, *n* = 70) or *Hepatozoon* (14%, *n* = 44). Consistent with our predictions, *Bartonella*, which has a relatively fast life-history, had higher average occupancy rate (0.56, range = 0.41–0.72) than *B. microti* (0.22, range = 0.13–0.37) and *Hepatozoon* (0.14, range = 0.00–0.35; Figure 3). In infected subpopulations, *Bartonella* had a similar prevalence (57%, range = 11–100%) to *B. microti* (48%, range = 6–100%) and *Hepatozoon* (33%, 9–100%).

All parasites showed high apparent turnover rates (Fig. 3). Intuitively, *I. ricinus*, as a host generalist, had high colonization (average = 0.50, range = 0.25–0.83) and low extinction rates (0.34, 0.09–0.54) compared to host-specialist parasites (Fig. 3C and D). Fleas and *I. trianguliceps* had similar apparent colonization rates (fleas: 0.24, 0.11–0.40; *I. trianguliceps*: 0.15, 0.04–0.26, Fig. 3C) and extinction rates (fleas: 0.61, 0.40–0.81; *I. trianguliceps*: 0.59, 0.00–1.00, Fig. 3D). As expected, *Bartonella* had higher colonization rates (0.41, 0.08–0.67) than both *B. microti* (0.10, 0.00–1.00) and *Hepatozoon* (0.07, 0.00–0.28), suggesting that life-history may affect metapopulation processes.

All parasites were highly effective at colonizing newly founded subpopulations. The observed colonization rates in new subpopulations were equal to or higher than expected colonization probabilities based on average infection probability and on the assumption that new subpopulations were founded by 2 voles (Table 2, S2.3). This is especially remarkable considering the time-lag between the vector's acquisition and transmission of certain microparasites.

**Table 2.** Comparison of estimated and observed colonization rates for new subpopulations

	Estimated total probability of colonization	Observed total colonization rate	Estimated mean probability of colonization	Observed mean colonization rate
Fleas	0.23	0.24 (*n* = 37)	0.22 (0.14–0.41)	0.19 (0.00–0.50)
*I. trianguliceps*	0.18	0.19 (*n* = 37)	0.15 (0.08–0.28)	0.12 (0.00–0.56)
*I. ricinus*	0.64	0.68 (*n* = 37)	0.62 (0.50–0.78)	0.62 (0.00–1.00)
*Bartonella*	0.54	0.41 (*n* = 22)	0.52 (0.38–0.66)	0.48 (0.00–1.00)
*B. microti*	0.20	0.26 (*n* = 23)	0.21 (0.12–0.36)	0.21 (0.00–0.50)
*Hepatozoon*	0.10	0.17 (*n* = 23)	0.11 (0.00–0.26)	0.22 (0.00–1.00)

*n* = Total number of new subpopulations trapped and sampled.

Estimated values were calculated under the assumption of two founders per subpopulation. Values represent the probability of at least one individual being infected, based on infection prevalence overall, or infection prevalence each year (mean probability of colonization). For mean estimated and observed colonization rates, ranges are given in brackets.

### Broad spatiotemporal patterns of infection probability (analysis I)

**Figure 4.**
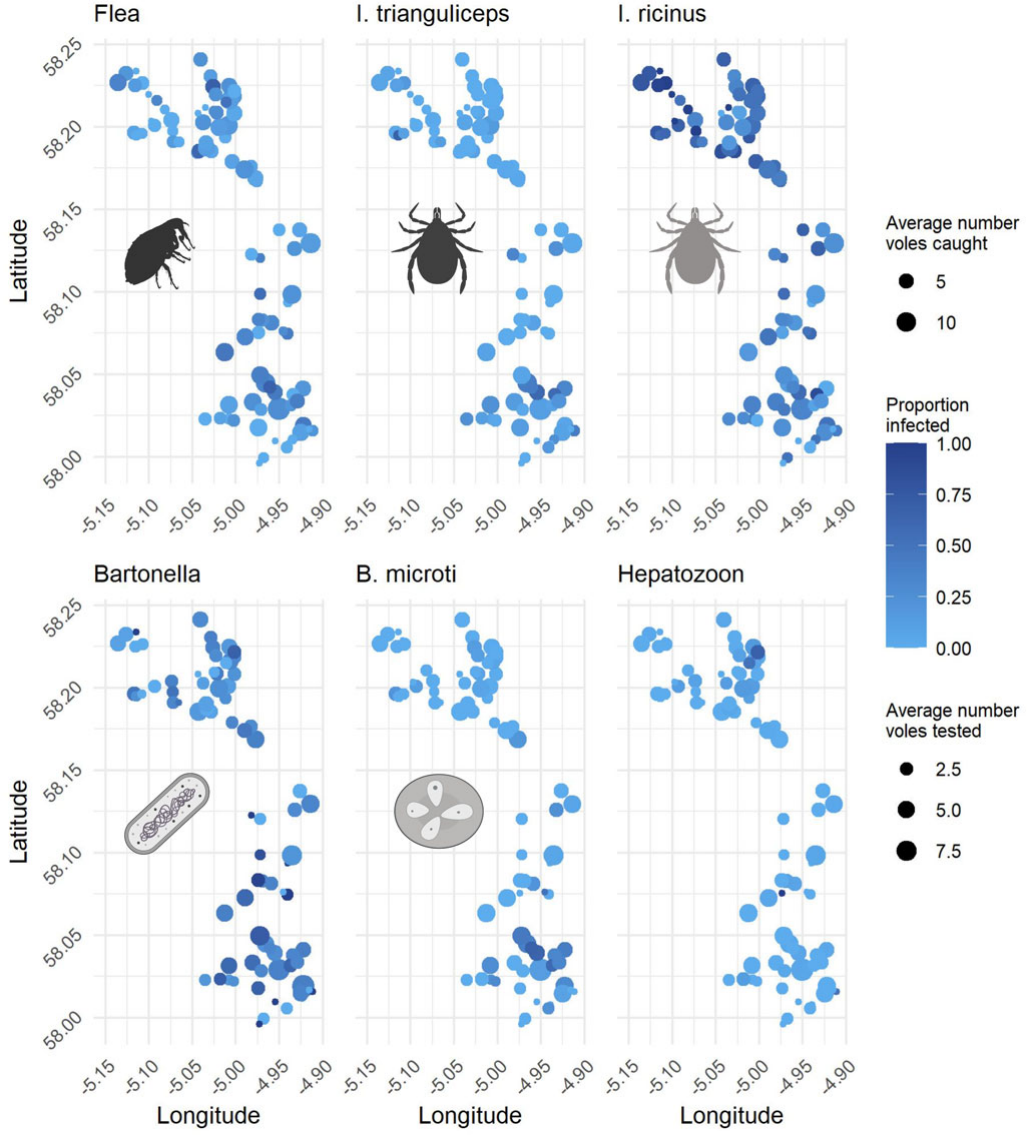
Maps showing the average proportion of animals infected across years. Bubble size indicates the average number of voles caught for ectoparasites and the average number of voles tested for microparasites. Only occupied and trapped subpopulations were included in the calculation. Created with BioRender.com.

Parasites showed distinct differences in spatiotemporal patterns (Fig. 4, Table 3). For *I. trianguliceps*, *I. ricinus* and the tick-borne *B. microti,* most variation in infection probability arose from patch-level differences (Table 3), suggesting that infection probability may be linked to the patch environment. *Ixodes trianguliceps* and *B. microti* were more prevalent in the south of the metapopulation (Fig. 4), while *I. ricinus* were common throughout metapopulation. Variations in infection probabilities of fleas and *Bartonella* arose between local subpopulations (Table 3), although some variation was attributed to patch identity. Indeed, there was no clear spatial structure for fleas and *Bartonella* (Fig. 4). For *Hepatozoon*, the variation between years was by far the greatest (Table 3); no *Hepatozoon* infection was detected in 2012. Some additional variation was associated with the patch identity, suggesting variations in *Hepatozoon* infections across the landscape (Table 3).

**Table 3.** Results of spatiotemporal variance analysis (analysis I), presented as the proportion of variance explained by each random effect, calculated using the mixedup package.

	Fleas	*I. trianguliceps*	*I. ricinus*	*Bartonella*	*B. microti*	*Hepatozoon*
PatchID	0.24	**0.80**	**0.50**	0.36	**0.97**	0.35
SubpopulationID	**0**.**64**	0.09	0.39	**0**.**62**	0.01	0.00
Year	0.12	0.11	0.11	0.02	0.02	**0**.**65**

For each parasite the highest proportion of variance explained by a random effect is highlighted in bold.

To highlight the salient results of GLMM analyses, we only present competitive models from lagged analyses (‘final model’). Results from each stage of analysis can be found in Supplementary Materials S3 (Tables S3.1–S3.3). Our initial predictions and final results (as presented below) are summarized in Supplementary Materials S4 (Table S4.1–S4.2).

### Spatiotemporal patterns of infection probability (analysis II & III)

All parasites showed local aggregation, with infection probability increasing with the contemporary local abundance of infected hosts (Table 4). At a larger spatial scale, only *I. trianguliceps* exhibited contemporary aggregation at a scale similar to vole dispersal distance (i.e., landscape-scale connectivity; Table 4), while flea, *Hepatozoon* and *I. ricinus* infection probability increased with the abundance of infected hosts at the metapopulation-scale, suggesting strong temporal patterns (Table 4).

**Table 4.** Coefficient estimates (odds ratio ± 85% confidence interval (CIs)) of best models from the analysis of scale-associated lagged infection patterns (analysis III)

	Variable	Fleas	*I. trianguliceps*	*I. ricinus*	*Bartonella*	*B. microti*	*Hepatozoon*
Current year	Local-scale infection abundance	**2.25 (1.96–2.58)**	**1.53 (1.25–1.88)**	**1.95 (1.71–2.23)**	**2.78 (2.23–3.47)**	**2.68 (1.84–3.88)**	**2.10 (1.67–2.65)**
	Landscape-scale infection connectivity		**1.88 (1.50–2.35)**			1.02* (0.71–1.47)	
	Metapopulation-scale infection abundance	**1.30 (1.14–1.50)**		**1.34 (1.19–1.51)**			**1.94 (1.38–2.71)**
Previous year	Local-scale infection abundance		**1.26 (1.04–1.52)**		**0.80 (0.66–0.98)**	**1.41 (1.14–1.73)**	
	Landscape-scale infection connectivity					**1.75 (1.23–2.50)**	
	Metapopulation-scale infection abundance			**0.79 (0.71–0.89)**	**0.80 (0.67–0.95)**		**0.52 (0.35–0.76)**
Individual	Weight		1.91 (1.54–2.38)		1.01 (0.83–1.24)	1.42 (1.12–1.80)	0.19 (0.10–0.37)
	Weight^2^	1.16 (1.01–1.33)	0.83 (0.70–0.99)		0.56 (0.46–0.69)		0.87 (0.55–1.36)
	Sex: female	0.06 (0.05–0.08)		0.62 (0.50–0.75)			
	Sex: male	0.12 (0.07–0.22)		1.02 (0.69–1.50)			
	Weight: sex: female	1.05 (0.84–1.31)		1.12 (0.98–1.29)			
	Weight: sex: male	1.23 (0.75–2.01)		1.58 (1.14–2.20)			
Model performance	Marginal *R*^2^	(0.22)	(0.29)	(0.16)	(0.27/0.26)	(0.26/0.20)	(0.58)
	Conditional *R*^2^	(0.25)	(0.43)	(0.28)	(0.36/0.36)	(0.55/0.46)	(0.58)

Informative parameters at local-, landscape and metapopulation-scales are presented in bold. Conditional *R*^2^ = variation explained by the full model including random effects; Marginal *R*^2^ = variation explained by fixed effects only.

*Parameter which was informative in the contemporary analysis but becomes uninformative in the lagged analysis.

As we predicted for ticks and tick-borne infections, the infection probabilities of both *I. trianguliceps* and *B. microti* were positively correlated with the local abundance of infected hosts in the previous year (Table 4). Further, for *B. microti* a competitive model suggested that infection probability was higher in areas with high infection connectivity in the previous year (Table 4).

At odds with expectations, *Bartonella* infection probability decreased with the local abundance of infections in the previous year (Table 4). Further, *Bartonella*, *Hepatozoon* and *I. ricinus* infection probability decreased following a year with a high metapopulation-scale abundance of infected hosts (Table 4). No lagged covariates were retained in the final models investigating fleas.

Models investigating spatiotemporal patterns described data well, with marginal pseudo-*R*^2^ of final models ranging from 0.16 for *I. ricinus* to 0.58 for *Hepatozoon* (Table 4). Intriguingly, for *Hepatozoon* the marginal and conditional *R*^2^ of the final model were the same, suggesting that lagged effects were able to account for a large amount of year-to-year variation in infection probability, previously accounted for by random effects (Table 4).

### Hosts and vectors as potential drivers of infection probability (analysis IV & V)

When considering contemporary and lagged drivers of ectoparasite infections, there was mixed evidence for infection probabilities correlating with host distribution. In all cases, fixed effects in best models explained little variation in infection risk, with low marginal R^2^ values of final models (*I. trianguliceps*: m*R*^2^ = 0.08; fleas: m*R*^2^ = 0.04, *I. ricinus*: m*R*^2^ = 0.06), suggesting that other drivers of ectoparasite dynamics, not considered in this study, may overshadow host effects. Indeed, random effects, capturing patch- and subpopulation-specific properties and year effects, explained most variance in ectoparasite infection probability (*I. trianguliceps*: c*R*^2^ = 0.51; fleas: c*R*^2^ = 0.33, *I. ricinus*: c*R*^2^ = 0.28).

*Ixodes trianguliceps* infection probability increased with contemporary metapopulation-scale abundance of hosts, with no evidence of host effects in the previous year (Table 5). At odds with our predictions, infection probability with fleas decreased with landscape-scale host connectivity in the previous year (Table 5). *Ixodes ricinus* infection probability decreased as local host abundance increased but increased in years of overall high metapopulation-scale host abundance.

**Table 5.** Coefficient estimates (odds ratio ± 85% CIs) of the best models from the lagged host-centred analysis (Analysis V) for ectoparasites only

	Variable	Fleas	*I. trianguliceps*	*I. ricinus*
Current year	Local-scale host abundance			**0.79 (0.66–0.94)**
	Landscape-scale host connectivity			
	Metapopulation-scale host abundance		**1.48 (1.16–1.89)**	**1.42 (1.20–1.69)**
Previous year	Local-scale host abundance			
	Landscape-scale host connectivity	**0.75 (0.60–0.94)**		
	Metapopulation-scale host abundance			
Individual	Weight		1.99 (1.59–2.49)	
	Weight^2^	1.15 (0.99–1.33)	0.81 (0.67–0.97)	
	Sex: female	0.05 (0.03–0.08)		0.49 (0.38–0.63)
	Sex: male	0.10 (0.05–0.20)		0.79 (0.50–1.23)
	Weight: sex: female	0.98 (0.78–1.23)		1.10 (0.96–1.27)
	Weight: sex: male	1.15 (0.69–1.91)		1.51 (1.08–2.11)
Model performance	Marginal *R*^2^	(0.04)	(0.08)	(0.06)
	Conditional *R*^2^	(0.33)	(0.51)	(0.28)

Informative parameters at local-, landscape and metapopulation-scales are presented in bold. conditional *R*^2^ = variation explained by the full model including random effects; marginal *R*^2^ = variation explained by fixed effects only as. * = parameter which was informative in the contemporary analysis but becomes uninformative in the lagged analysis.

When considering drivers of microparasite infection probability, *B. microti* and *Bartonella* were both correlated with vector variables. However, only *Bartonella* was additionally correlated with host distribution at several spatial and temporal scales. *B. microti* infection probability increased with high contemporary local *I. trianguliceps* abundance and high *I. trianguliceps* landscape connectivity (Table 6). Additionally, infection probability increased with lagged local *I. trianguliceps* abundance. Non-intuitively if *I. ricinus* contributed to *B. microti* transmission, *B. microti* infection probability decreased with lagged local *I. ricinus* abundance (Table 6). The final best model described infection probability with *B. microti* well, with an m*R*^2^ of 0.26 (Table 6).

**Table 6.** Coefficient estimates (odds ratio ± 85% CIs) of the best models from the lagged host and vector-centred analysis (analysis V) for microparasites only

	Variable	Scale	*Bartonella*	*B. microti*	*Hepatozoon*
Current year	Host	Local-scale abundance			
		Landscape-scale connectivity	**0.59 (0.44–0.78)**		
		Metapopulation-scale abundance			**4.05 (2.55–6.41)**
	Fleas	Local-scale abundance		/	
		Landscape-scale connectivity	**1.49 (1.18–1.87)**	**/**	
		Metapopulation-scale abundance		**/**	**0.76 (0.43–1.37)**
	*I. trianguliceps*	Local-scale abundance	**/**	**1.45 (1.05–1.99)**	
		Landscape-scale connectivity	**/**	**1.68 (1.20–2.36)**	
		Metapopulation-scale abundance	**/**		
	*I. ricinus*	Local-scale abundance	**/**		
		Landscape-scale connectivity	**/**		
		Metapopulation-scale abundance	**/**		
Previous year	Host	Local-scale abundance			
		Landscape-scale connectivity	**1.44 (1.14–1.83)**		
		Metapopulation-scale abundance			
	Fleas	Local-scale abundance			
		Landscape-scale connectivity	**1.32 (1.07–1.63)**		
		Metapopulation-scale abundance			**0.59 (0.43–0.79)**
	*I. trianguliceps*	Local-scale abundance		**1.83 (1.30–2.57)**	
		Landscape-scale connectivity			
		Metapopulation-scale abundance			
	*I. ricinus*	Local-scale abundance		**0.66 (0.46–0.96)**	
		Landscape-scale connectivity			
		Metapopulation-scale abundance			**0.36 (0.24–0.54)**
Individual	Weight		0.97 (0.82–1.14)	1.35 (1.08–1.68)	0.29 (0.20–0.44)
	Weight^2^		0.57 (0.48–0.67)		1.18 (0.86–1.61)
Model performance	Marginal *R*^2^		(0.12/0.12)	(0.26)	(0.51)
	Conditional *R*^2^		(0.35/0.36)	(0.48)	(0.54)

Informative parameters at local-, landscape and metapopulation-scales are presented in bold. Conditional *R*^2^ = variation explained by the full model including random effects; marginal *R*^2^ = variation explained by fixed effects only as. * = parameter which was informative in the contemporary analysis but becomes uninformative in the lagged analysis.

Similarly, *Bartonella* infection probability increased with flea connectivity at the landscape-scale in both the contemporary and previous years (Table 6). In addition, infection probability decreased with host connectivity in the current year but increased with host connectivity in the previous year (Table 6). The 2 competing models described relatively little variation in the infection probability of *Bartonella*, with an m*R*^2^ of 0.12.

Drivers of *Hepatozoon* probability were obscured by strong annual effects. *Hepatozoon* infection probability increased with high metapopulation-scale host abundance (Table 6). Additionally, infection probability decreased with high metapopulation-scale fleas and *I. ricinus* abundance in the previous year (Table 6). The *R*^2^ of lagged models exploring *Hepatozoon* was very high (m*R*^2^ = 0.51, c*R*^2^ = 0.54), suggesting that models mainly explain the year-to-year variation in *Hepatozoon* infection probability.

## Discussion

Empirical studies have investigated spatiotemporal dynamics of directly transmitted parasites in spatially structured host–parasite systems (e.g., *Melitaea cinxia* butterflies and *Cotesia melitaearum* parasitoid: Lei and Hanski, 1997; *Plantago lanceolata* plants and *Podosphaera plantaginis* rust pathogen: Laine and Hanski, 2006; fungal pathogens in the forest herb *Anemone nemorosa*: Van Dijk *et al*., 2022), while the complex effects of host–vector–parasite interactions on parasite dynamics have largely been overlooked. In this study, we compared several host–vector–parasite systems on the backdrop of a classic metapopulation, marked by small population sizes, frequent dispersal and high turnover rates of hosts and parasites. Despite being correlative rather than demonstrating causality, interesting general patterns emerged and generated hypotheses which may inform future experimental studies, should such studies become feasible on a landscape-scale. We found evidence of spatial correlation of infection probability with infected conspecifics in the same local subpopulation and, for *I. trianguliceps* and *B. microti*, of connectivity to conspecifics in the wider landscape. Microparasite infection probability was strongly correlated with vectors and in the case of *Bartonella*, host distributions, at local- and landscape-scales. In contrast, we found mixed evidence of direct host effects at either scale on ectoparasite infection probability. With a few exceptions, spatial scales of infection patterns and driver effects were consistent with the hypothesis that the connectivity of landscapes is mediated by host-dispersal distances. Lastly, we found some evidence, that parasite dynamics including occupancy and colonization rates across the metapopulation are associated with the life-history of ectoparasites and microparasites.

### Parasite persistence on the edge of extinction

As expected, water vole hosts and parasites experienced high degrees of turnover. Intriguingly, the apparent colonization rates of most parasites were surprisingly high. This is best illustrated when considering newly established host subpopulations. All parasites were likely to colonize a new subpopulation in the first year, rather than gradually arriving in the subpopulation. Similar patterns have been observed for parasites able to disperse independently from their host (Lei and Hanski, 1997, 1998); however, this observation was unexpected for parasites wholly relying on their host and vectors for dispersal and transmission. This is especially surprising for parasites like ticks that spend a short proportion of their lifespan on their host and hence have limited dispersal and transmission opportunities.

The ability of some ectoparasites to survive for a time without the host by entering diapause may allow them to almost instantaneously colonize new host subpopulations. Nidicolous ixodid ticks, including *I. trianguliceps* (Randolph, 2004), are known to resist starvation in the absence of their host for years, by entering a developmental diapause during which the last blood meal is retained undigested (Gray *et al*., 2013). If we assume that tick-borne microparasites are retained during this process, microparasites would be able to infect any new hosts almost instantaneously. In contrast, while some flea species undergo diapause, as discussed in Krasnov (2008), it is unknown whether *Bartonella* infections are retained during diapause as adults. Flea larvae can be infected with *Bartonella* from adult flea feces or gut voids and will develop into infected adult fleas (Morick *et al*., 2013). Thus, environmental contamination may serve as a temporal reservoir of *Bartonella* infection in abandoned burrows (Gutiérrez *et al*., 2015). However, the length of time *Bartonella* remains infective within the burrow is unknown and it seems unlikely that environmental sources could lead to reinfection after several months or years of burrow abandonment. This could explain why *Bartonella* had a lower colonization rate than expected. The effect of vector survival in the absence of suitable hosts in stochastic systems is an interesting avenue for further disease metapopulation research.

Similarly, high colonization rates could be observed if dispersers are disproportionately infected by parasites. For example, studies suggest that juveniles have higher infection probabilities of *Bartonella* than adults (Telfer *et al*., 2007; Paziewska *et al*., 2012), likely due to higher mobility of juveniles and/or acquired immunity in later life. As Sutherland *et al*. (2012) suggest that dispersal is undertaken by water vole juveniles, if juveniles have higher parasite prevalence, colonization rates may increase.

### Weak direct host effects on ectoparasite infection probability

We expected a positive effect of local host abundance and landscape-scale connectivity on the infection probability of rodent-associated ectoparasites. In contrast, *I. trianguliceps* infection probability increased with high host abundance at the metapopulation-scale in the current year, and flea infection probability decreased in areas with high host connectivity in the previous year. These findings were completely unexpected, and it is unclear whether these patterns resulted from actual drivers, or whether they resulted from a correlation due to environmental factors.

*Ixodes ricinus* infection probability decreased in larger subpopulations, suggesting a dilution effect, where at high host densities a limited number of *I. ricinus* are divided between more hosts. Similar patterns have previously been recorded for other ectoparasites (Telfer *et al*., 2007). Further, as *I. ricinus* abundance can be related to the presence of deer and sheep (Gilbert *et al*., 2012), and the effect of water voles on *I. ricinus* abundance is likely relatively small, the risk of parasitism may be very high in an area despite low vole abundances and vice versa.

Notably, for all ectoparasites, host variables described very little variation in ectoparasite infections (low *R*^2^), raising the potential for other drivers of ectoparasite dynamics overshadowing potential host effects. Alternative hosts may reduce (Telfer *et al*., 2005; Renwick and Lambin, 2013) or increase infection probability (Telfer *et al*., 2005) of parasites within populations, depending on their host competency and relative abundance in a community (Dobson, 2004; Fenton *et al*., 2015). In metapopulation systems, where local host populations are small and prone to stochastic extinction, these effects are likely amplified. Additionally, alternative hosts may alter the connectivity of a landscape which parasites experience (Renwick and Lambin, 2013); for example, studies investigating the epidemiology of plague in prairie dog metapopulations suggested that white-footed mice increased landscape-scale connectivity by moving infected vectors between susceptible prairie dog coteries (Salkeld *et al*., 2010; George *et al*., 2013).

In the Assynt metapopulation, field voles (*Microtus agrestis*) are alternative hosts of fleas, *Bartonella,* and less frequently *I. trianguliceps* and *B. microti* (Davies, 2014). Field voles occupy water vole habitat, utilizing water vole runs and burrows, suggesting a potential avenue for cross-species transmission. Further, field voles inhabit patches of grassland habitat unsuitable for water voles. Thus, local field vole abundance and distribution between water vole habitat patches may affect parasite patterns across the metapopulation. Moreover, field voles show population cycles of approximately 3 years (Lambin *et al*., 2000). This cyclical rise in the abundance of alternative hosts may explain some of the temporal variation observed in parasite dynamics, especially fleas. Future research could help elucidate the potential role of alternative hosts in the transmission and persistence of parasites in spatially heterogeneous and stochastic host populations.

Additionally, ectoparasite vectors spend considerable time ‘off-host’, where environmental conditions may affect their survival (Manlove *et al*., 2022). The probability of flooding (Ulmanen, 1972), the vegetation used for nesting, soil type and hence burrow depth (Krasnov *et al*., 1996, 2021) have all been shown to affect the distribution, survival and development of nest-dwelling arthropods, in particular fleas. Indeed, our spatiotemporal variance analysis (*Analysis I*), where patch identity was found to affect the infection probability of all parasites, may reflect the influence of local environmental conditions on spatial dynamics of ecto- and microparasites.

### The relative importance of hosts *vs* vectors in microparasite dynamics reflects microparasite life-history

Few studies have investigated the relative influence of hosts *vs* vectors on microparasite dynamics (Smith *et al*., 2005; Telfer *et al*., 2007; Rodríguez-Pastor *et al*., 2019), and we lack knowledge on how host and vector dynamics interact in metapopulation systems. As hosts are vital for the dispersal of vectors in metapopulations, we expected the dynamics of vector-borne microparasites to be correlated with both hosts and vectors locally and at the landscape-scale. Yet, results suggest that drivers of microparasite patterns across the metapopulation may differ between the 2 main vector-borne microparasites: *Bartonella* was associated with both host and vector dynamics. In contrast, *B. microti* was positively correlated with vector dynamics, while no consistent association with host dynamics was found.

In a recent perspective, Manlove *et al*. (2022) suggested that spatial transmission dynamics of directly transmitted parasites are influenced by the parasite's life-history. Where pathogens persist in the environment for a considerable amount of time, instances of transmission should be concentrated where hosts encounter the environmental stage of the pathogen, while host abundance and connectivity may be secondary in determining spatial patterns of infection probability (Manlove *et al*., 2022). While the complexity of vector-borne parasites is not addressed by Manlove, arthropod vectors are analogous to the ‘environment’, in which the parasite must survive until onward transmission. Reflecting their life-histories, our results suggest that for vector-borne microparasites, vector dynamics may shape spatial and temporal patterns in infection probability, adding several layers of complexity. However, only for *Bartonella*, which has a fast life-history and for which transmission from vector to host occurs relatively rapidly, did we find evidence for an additional host effect on infection probability. In contrast, for *B. microti*, which survives for several months or even years within its tick vector before onward transmission (Jalovecka *et al*., 2019), vector variables outperformed host effects. Our findings support the assertion that the relative importance of hosts *vs* vectors for microparasite infection probability may be governed by the relative time spent in each ‘environment’. To understand the generality of these findings, future studies should collect empirical data on the importance of vector dynamics, contrasting host–vector–parasite systems with different transmission dynamics (e.g., independently mobile vectors such as mosquitoes, parasites that spend more or less time in host *vs* vector-environment, etc.).

### Connectivity of metapopulation mediated by the dispersal distance of the focal host

Parasite infection patterns across the metapopulations supported the hypothesis that connectivity and spatial isolation in a metapopulation are mediated by the dispersal distance of the main host. We contrasted rodent-associated parasites with the dynamics of *I. ricinus*, a generalist tick, known to frequently infect sheep and deer, which roam over vast distances. Accordingly, *I. ricinus* was more homogeneously distributed across the metapopulation, with high infection rates, low extinction probability and high colonization rates. In contrast, rodent-associated parasites, especially *B. microti* and *I. trianguliceps*, showed a more aggregated distribution. Further, the infection probabilities of most rodent-associated parasites were correlated with landscape-scale connectivity of hosts and vectors rather than metapopulation-scale abundances. These findings are consistent with several studies that showed that the dispersal and spread of pathogens and parasites are shaped by the realized dispersal ability of hosts. Using genetic analysis, McCoy *et al*. (2003) demonstrated that *Ixodes uriae* ticks associated with 2 hosts with different dispersal abilities showed different genetic isolation between populations. *Ixodes uriae* infecting kittiwakes, the stronger disperser, showed less genetic isolation across populations than ticks infecting puffins, the weaker disperser. Further, Watts *et al*. (2018) showed that the potential spread of *Borrelia* spp. and its associated vectors *Ixodes scapularis* was mediated by the effective dispersal distance of the different main hosts of *I. scapularis* life stages.

Contrary to the other rodent-associated parasites, fleas and *Bartonella* distributions were not aggregated at the landscape-scale when investigating scale-associated contemporary infection patterns (analysis II). Flea species were pooled while *Bartonella* infections were analysed at the genus level. Thus, divergent species-specific patterns may have blurred any overall pattern. Further, we underestimated true flea prevalence and abundance by only enumerating fleas brushed from trapped voles. Moreover, connectivity was calculated based on trapped and sampled neighbouring patches, further increasing the likelihood of false negatives. As with the estimation of dispersal by water voles, the presence of false negatives necessarily leads to an overestimation of the scale of connectivity, as nearby potential sources of colonists are overlooked and estimated dispersal, therefore, calls upon more distant colonists (Sutherland, 2013).

### Life-histories may affect infection dynamics in a metapopulation setting

We provide evidence supporting the hypothesis that parasite life-history may affect metapopulation dynamics. *Bartonella* had higher occupancy rates and colonization rates across the metapopulation than *B. microti* or *Hepatozoon* microparasites. This is likely due to the way life-history traits affect the transmission rates of the parasites. As fleas feed successively on several hosts, the transmission rate of *Bartonella* is likely higher compared to the tick-vectored *B. microti*, which can only infect 1 host per infected tick. Such life-history-related patterns were also observed by van Dijk *et al*. (2022) for several plant pathogens infecting a metapopulation of the forest herb *A. nemorosa.* Pathogens that transmitted between plants *via* spores had higher patch occupancy, within patch prevalence and colonization rates, than pathogens that caused systemic, perennial infections in rhizomes. Other parasite traits may additionally affect transmission rates to produce the patterns found. Differing infectious periods of the 2 microparasites may further explain the patterns observed. While *B. microti* is assumed to be chronic in most rodent species, some laboratory studies suggest that transmission can only occur during the acute period of infection, which lasts several days to weeks (Randolph, 1995; Gray *et al*., 2002). If this is the case for rodents in natural populations is unclear. In comparison, bacteriaemia for *Bartonella* lasts for several weeks (Birtles *et al*., 2001; Birtles, 2005), potentially reflecting longer infectious periods. A shorter infectious period may lead to fewer successful dispersal events for the parasite, and thus lower occupancy and colonization rates (Cross *et al*., 2005). Further, immunity or resistance to the parasite in the population may affect transmission and metapopulation rates. If the abundance of susceptibles is lower due to immunity, either through natural resistance or acquired immunity after infection, local parasite prevalence, colonization rates and overall occupancy rates may be lower, and extinction rates higher than expected (Lloyd-Smith *et al*., 2005). There is some empirical evidence that some rodent species acquire immunity against some *Bartonella* species (Birtles *et al*., 2001; Sherlock *et al*., 2013). Our findings suggest that various life-history traits of parasites may play an important role in shaping spatiotemporal patterns of infection and should be considered. In contrast to our expectations, no such patterns were observed for ectoparasites, possibly as flea presence and abundance were underestimated with the methods used.

Contrary to our initial predictions, both short- and long-lived vectors and their associated microparasites were related to infection dynamics and/or drivers in the previous year. Delays of 12–24 months between tick population dynamics and tick-borne infections are not uncommon (Laurenson *et al*., 2003; Aminikhah *et al*., 2021), and are in line with the long delays in transmission introduced by tick-feeding behaviour, moults and diapause. Conversely, previous research has indicated that the lag between flea prevalence and flea-borne infections is shorter, usually around 1–6 months (refer to Smith *et al*., 2005; Telfer *et al*., 2007), likely introduced by a short overwinter diapause experienced by fleas in temperate regions. Our findings suggest that longer lagged effects are possible even for short-lived vectors and their microparasites and should be considered.

## Conclusions

Our study offers a unique insight into vector-borne parasites with different life-histories in a classic metapopulation setting. It suggests that any future comprehensive theory of vector-borne microparasite persistence in metapopulations must consider several layers of complexity. This includes, but is not restricted to, the relative effect of hosts and vectors at multiple spatial scales and the effect of life-history of vectors and microparasites on spatiotemporal patterns in host populations.

## Data Availability

Data available on Open Science Framework (OSF): https://doi.org/10.17605/OSF.IO/PYQSN.

## Supplementary material

For supplementary material accompanying this paper visit https://doi.org/10.1017/S0031182023000677. click here to view supplementary material
