# Supplementary material for: Patterns and drivers of vector-borne microparasites in a classic metapopulation
Source: Parasitology. 2023 Jul 31;150(10):866–82. doi: 10.1017/S0031182023000677 (PMC10577662; doi:10.1017/S0031182023000677)
Supplement: Supplementary file 1 [file S0031182023000677sup.zip › S0031182023000677sup001.docx]

| **here Parasite name** | **Host range** | **Host encounter** | **Feeding behaviour** | **Life cycle** | **Length of infestation** |
| --- | --- | --- | --- | --- | --- |
| Flea spp. | Small to medium-sized burrowing mammals  Host specificity varies between flea species,  most fleas are associated with one or few principal hosts and some auxiliary hosts (Krasnov *et al.*, 2003, 2004) | Within a host nest or burrow  Direct transmission between host individuals during physical encounters | Several blood meals as an adult | Weeks-months  Several generations are produced per year  Seasonal cycles of reproduction and infection rates found for several flea species infecting voles in the UK  (Telfer *et al.*, 2007; Oliver *et al.*, 2009)  Longevity is influenced by environmental conditions and the host lifecycle  Reproductive diapause as adults (Krasnov, 2008) observed for several species  Diapause in egg and pupal stages observed for fleas infecting rabbits in NE Spain (Osácar *et al.*, 2001) | Unknown  Time spent on the host vs burrows or nests can vary between species of flea, host and season (reviewed in Krasnov, 2008)    Minutes – days per feed depending on species, host, age and season |
| *Ixodes trianguliceps* | Small mammals | Nidicolous, hosts nests and burrows  Some indications that larvae are exophilic | Once per life stage  (Randolph, 2004) | 2-5 years (Randolph, 1995, 2004)  Moults off-host  The time between moults (21-250 days)  Seasonal cycles well documented  Developmental and behavioural diapause  (Randolph, 1975) | Larvae: ~ 7 days  Nymphs: ~ 9.5 days  Adult females: 12-17 days  (Randolph, 1975) |
| *Ixodes ricinus* | Host generalist:  Small mammals, birds, lizards, and larger mammals (cattle, deer) | All stages exophilic  Questing behaviour in vegetation | Once per life stage  (Randolph, 2004) | 2-3 years  Moults off-host  Seasonal cycles well documented  Developmental and behavioural diapause documented in Scotland | Adult females: ~6-8 days  Up to 30 days observed  (Campbell, 1948) |

| **Parasite name** | **Vertebrate hosts** | **Invertebrate hosts/Vectors** | **Transmission mode** | **Transstadial/transovarial transmission in vector** | **Length of infection (mammal host)** |
| --- | --- | --- | --- | --- | --- |
| Rodent-specific *Bartonella spp.* | Rodents | Arthropods  Fleas are known to be vectors and potential reservoirs (Birtles, 2005; Gutiérrez et al., 2015)  *I. ricinus* is indicated as a potential vector but transmission is not yet proven (Król *et al.*, 2021) | Flea-borne | No transovarial transmission  Alternative vertical non-transovarial transmission  (Morick *et al.*, 2013) | Acute  Bacteraemia lasts from a few weeks to several months, with evidence of recrudescence in some wild rodents  (Birtles *et al.*, 2001) |
| *Babesia microti (Babesia sensu lato)* | Rodents | *Ixodes* ticks (Jalovecka *et al.*, 2019)  *I. trianguliceps* main vector in natural rodent populations (Bown *et al.*, 2008) | Tick-borne | Transstadial transmission proven  Unlikely to persist in vector for more than one moult (Gray *et al.*, 2002)    No transovarial transmission (Jalovecka *et al.*, 2019) | Chronic  Laboratory studies suggest that transmission to ticks is only possible in the acute phase (several days); no evidence for this in wild rodents (Randolph, 1995; Gray *et al.*, 2002) |
| *Hepatozoon spp.* | Reptiles (Tomé *et al.*, 2013)  Mammals and birds (Smith, 1996) | DNA detected in fleas, tick, lice and mite spp. Fleas are indicated as the main invertebrate host for rodent-associated Hepatozoon (Rigó *et al.*, 2016)  Invertebrates are the definitive host (Smith, 1996) | Ingestion of infected definitive host | Some species are transstadially and transovarially transmitted | Unknown |

**References**

**Birtles, RJ, Hazel, SM, Bennett, M, Bown, K, Raoult, D and Begon, M** (2001). Longitudinal monitoring of the dynamics of infections due to Bartonella species in UK woodland rodents. *Epidemiology and Infection* **126**, 323–329. doi: 10.1017/S095026880100526X.

**Bown, KJ, Lambin, X, Telford, GR, Ogden, NH, Telfer, S, Woldehiwet, Z and Birtles, RJ** (2008). Relative importance of Ixodes ricinus and Ixodes trianguliceps as vectors for Anaplasma phagocytophilum and Babesia microti in field vole (Microtus agrestis) populations. *Applied and Environmental Microbiology* **74**, 7118–7125. doi: 10.1128/AEM.00625-08.

**Campbell, JA** (1948). The life history and development of the sheep tick Ixodes ricinus Linnaeus in Scotland, under natural and controlled conditions.

**Gray, J, von Stedingk, LV, Gurtelschmid, M and Granstrom, M** (2002). Transmission Studies of Babesia microti in Ixodes ricinus Ticks and Gerbils. *Journal of clinical microbiology* **40**, 1259–1263.

**Jalovecka, M, Sojka, D, Ascencio, M and Schnittger, L** (2019). Babesia Life Cycle - When Phylogeny Meets Biology. *Trends in Parasitology* **35**, 356–368.

**Krasnov, B** (2008). *Functional and Evolutionary Ecology of fleas: A model for ecological parasitology*. Cambridge University Press.

**Krasnov, BR, Sarfati, M, Arakelyan, MS, Khokhlova, IS, Burdelova, N v. and Degen, AA** (2003). Host specificity and foraging efficiency in blood-sucking parasite: Feeding patterns of the flea Parapulex chephrenis on two species of desert rodents. *Parasitology Research* **90**, 393–399. doi: 10.1007/s00436-003-0873-y.

**Krasnov, BR, Poulin, R, Shenbrot, GI, Mouillot, D and Khokhlova, IS** (2004). Ectoparasitic “jacks-of-all-trades”: Relationship between abundance and host specificity in fleas (Siphonaptera) parasitic on small mammals. *American Naturalist* **164**, 506–516. doi: 10.1086/423716.

**Król, N, Militzer, N, Stöbe, E, Nijhof, AM, Pfeffer, M, Kempf, VAJ and Obiegala, A** (2021). Evaluating transmission paths for three different bartonella spp. In ixodes ricinus ticks using artificial feeding. *Microorganisms* **9**, 1–12. doi: 10.3390/microorganisms9050901.

**Morick, D, Krasnov, BR, Khokhlova, IS, Gutiérrez, R, Gottlieb, Y and Harrus, S** (2013). Vertical nontransovarial transmission of Bartonella in fleas. *Molecular Ecology* **22**, 4747–4752. doi: 10.1111/mec.12408.

**Oliver, MK, Telfer, S and Piertney, SB** (2009). Major histocompatibility complex (MHC) heterozygote superiority to natural multi-parasite infections in the water vole (Arvicola terrestris). *Proceedings of the Royal Society B* **276**, 1119–1128. doi: 10.1098/rspb.2008.1525.

**Osácar, JJ, Lucientes, J, Calvete, C, Peribañez, MA, Gracia, MJ and Castillo, JA** (2001). Seasonal Abundance of Fleas (Siphonaptera: Pulicidae, Ceratophyllidae) on Wild Rabbits in a Semiarid Area of Northeastern Spain. *Journal of Medical Entomology* **38**, 405–410.

**Randolph, SE** (1975). Seasonal Dynamics of a Host-Parasite System: Ixodes trianguliceps (Acarina: Ixodidae) and its Small Mammal Hosts. *The Journal of Animal Ecology* **44**, 425–449. doi: 10.2307/3605.

**Randolph, SE** (1995). Quantifying parameters in the transmission of Babesia microti by the tick Ixodes trianguliceps amongst voles ( Clethrionomys glareolus). *Parasitology* **110**, 287–295. doi: 10.1017/S0031182000080872.

**Randolph, SE** (2004). Tick ecology : processes and patterns behind the epidemiological risk posed by ixodid ticks as vectors. *Parasitology* **129**, pp.37-65. doi: 10.1017/S0031182004004925.

**Rigó, K, Majoros, G, Szekeres, S, Molnár, I, Jablonszky, M, Majláthová, V, Majláth, I and Földvári, G** (2016). Identification of Hepatozoon erhardovae Krampitz, 1964 from bank voles (Myodes glareolus) and fleas in Southern Hungary. *Parasitology Research* **115**, 2409–2413. doi: 10.1007/s00436-016-4992-7.

**Smith, TG** (1996). The Genus Hepatozoon (Apicomplexa : Adeleina). *The Journal of Parasitology* **82**, 565–585.

**Telfer, S, Begon, M, Bennett, M, Bown, KJ, Burthe, S, Lambin, X, Telford, G and Birtles, R** (2007). Contrasting dynamics of Bartonella spp. in cyclic field vole populations: The impact of vector and host dynamics. *Parasitology* **134**, 413–425. doi: 10.1017/S0031182006001624.

**Tomé, B, Maia, JPMC and Harris, DJ** (2013). Molecular Assessment of Apicomplexan Parasites in the Snake Psammophis from North Africa: Do Multiple Parasite Lineages Reflect the Final Vertebrate Host Diet? . *Journal of Parasitology* **99**, 883–887. doi: 10.1645/12-95.1.
