# Supplementary material for: Patterns and drivers of vector-borne microparasites in a classic metapopulation
Source: Parasitology. 2023 Jul 31;150(10):866–82. doi: 10.1017/S0031182023000677 (PMC10577662; doi:10.1017/S0031182023000677)
Supplement: Supplementary file 1 [file S0031182023000677sup.zip › S0031182023000677sup002.docx]

**S2. Additional Methods**

**S2.1 Microparasite detection: Apicomplexan-specific nested PCR**

*B. microti* was detected using a nested PCR, targeting an Apicomplexan-specific 18S rRNA fragment (Simpson *et al*., 2005). Primers (Table 1) were prepared following manufacturer instructions to a 10 µmol/µL concentration. A 2.5 µL aliquot of DNA was added to the first-round reaction.  The first-round MasterMix included 12.5 µL 2xMyTaq^TM^Red Mix (Bioline), 6 µL sterilised distilled water and 2 µL of each primer (BMF1 and BMR1, Eurofins genomics). The thermocycle consisted of a 3-minute activation stage at 94˚C, followed by 40 cycles of 30 seconds at 94˚C, 30 seconds at 55˚C and 30 seconds at 72˚C.

A 1µL aliquot of the first-round product was further amplified. Volumes of 2xMyTaq^TM^Red Mix and primers (BMF2 and BMR2, Eurofins genomics, see Appendix) were kept consistent with round one, increasing water to 7.5µL. The thermocycle was kept consistent with the first round. DNA fragments were visualised by gel electrophoresis on a 3% agarose gel at 145V for 1h.

**Table S2.1** Primer-sequences (provider: Eurofins Genomics) used in nested PCR targeting Apicomplexan-specific 18s rRNA fragment (Simpson et al., 2005).

| **Oligo name** | **Sequence (5’ → 3’)** |
| --- | --- |
| BMF1 | GCGATGTATCATTCAAGTTTGTG (23) |
| BMR1 | TGTTATTGCCTTACACTTCCTTGC (24) |
| BMF2 | ACGGCTACCACATCTAAGGAAGGC (24) |
| BMR2 | TCTCTCAAGGTGCTGAAGGA (20) |

**S2.2 Microparasite detection: Bartonella genus-specific qPCR**

*Bartonella* infection was identified using a genus-specific qPCR, targeting a fragment of the *rnp*-gene in samples from 2012-2019. A 2.5µl DNA sample was used in a final volume of 10 µl. The reaction mix included 5 µl SensiFAST™ Probe No-ROX (Bioline, UK), 0.9 µM of the forward and reverse primer, rnpBR2 and rnpBF2 (see Appendix), 0.125 µM rnpB- probe, 0.5 µl MgCl_2_ Solution (50mM) (Bioline, UK) and 0.8 µl H_2_O. The thermocycle consisted of a 2-minute polymerase activation stage at 94˚C, followed by 50 cycles of denaturation at 94°C for 20 seconds and annealing and extension at 59°C for 40 seconds.

**Table S2.2:** Primer-sequences (provider: Eurofins Genomics) used in nested PCR targeting rnp fragment (Hassall, 2020)

| **Oligo name** | **Sequence (5’ → 3’)** |
| --- | --- |
| rnpBR2 | CACCYTTTCACCCTTACC |
| rnpBF2 | AGGAAAGTCCGGGCTC |
| Dual-labelled probe  (5’- FAM, 3’-TAMRA) | ACCCTAGGGAAAGTCCCACAGAAAG |

**S2.3 A worked example of calculating the expected parasite colonisation rate in newly colonised subpopulations.**

The expected colonisation rate in newly colonised subpopulations was calculated with the assumption that each subpopulation requires two new founders (usually one male, one female) to become established:

$$Pr(X|p)=1-\left( 1-p \right)^{2}$$

Where *p* is the parasite prevalence each year and Pr(X|*p*) is the probability that at least one of the founders is infected with the parasite. For example, *I. trianguliceps* prevalence was 0.07 across the metapopulation in 2012.

$$\Pr\left( X | 0.07 \right)=1-\left( 1-0.07 \right)^{2}=0.1351$$

Therefore, the expected colonisation rate of a new subpopulation with two new founders was approximately 0.135. the table below calculated the *I. trianguliceps* expected colonisation rate each year:

| **Year** | **Prevalence (p)** | **Expected colonization rate (**$\mathbf{Pr}\left( \mathbf{X} \vert\mathbf{p} \right)$ |
| --- | --- | --- |
| 2012 | 0.07 | 0.14 |
| 2013 | 0.15 | 0.28 |
| 2014 | 0.1 | 0.19 |
| 2015 | 0.05 | 0.10 |
| 2016 | 0.06 | 0.12 |
| 2017 | 0.1 | 0.19 |
| 2018 | 0.04 | 0.08 |
| 2019 | 0.05 | 0.10 |

Therefore, the average expected colonisation rate for I. trianguliceps was 0.15 (range = 0.08-0.28).

**S3: Additional Results**

**Table S3.1** Coefficients estimates (Odds ratio +/- 85% CIs) of best models from the analysis of scale-associated contemporary infection patterns (*Analysis II*). Informative parameters are presented in bold. Conditional R^2^ = variation explained by the full model including random effects; Marginal R^2^ = variation explained by fixed effects only.

| Stage | Variable | Fleas | *I. trianguliceps* | *I. ricinus* | *Bartonella* | *B. microti* | *Hepatozoon* |
| --- | --- | --- | --- | --- | --- | --- | --- |
| Current year | Local-scale infection abundance | **2.21 (1.96-2.49)** | **1.65 (1.41-1.93)** | **2.04 (1.81-2.30)** | **2.67 (2.23-3.19)** | **1.86 (1.53-2.26)** | **1.94 (1.64-2.30)** |
|  | Landscape-scale infection connectivity |  | **1.52 (1.28-1.79)** |  |  | **1.42 (1.11-1.82)** | **1.32 (1.03-1.71)** |
|  | Metapopulation-scale infection abundance | **1.29 (1.14-1.45)** | **1.46 (1.27-1.68)** | **1.24 (1.09-1.41)** |  |  | **1.53 (1.04-2.26)** |
| Individual | Weight |  | 1.80 (1.53-2.12) |  | 1.07 (0.93-1.23) | 1.83 (1.54-2.19) | 0.42 (0.33-0.54) |
|  | Weight² | 1.18 (1.06-1.31) | 0.82 (0.72-0.93) |  | 0.57 (0.49-0.66) |  | 1.25 (1.00-1.55) |
|  | Sex:Female | 0.06 (0.05-0.08) |  | 0.68 (0.55-0.83) |  |  |  |
|  | Sex:Male | 0.13 (0.08-0.21) |  | 1.10 (0.76-1.58) |  |  |  |
|  | Weight:Sex:Female | 0.94 (0.79-1.12) |  | 1.22 (1.09-1.37) |  |  |  |
|  | Weight:Sex:Male | 1.19 (0.81-1.76) |  | 1.66 (1.26-2.19) |  |  |  |
| Model performance | **Marginal R²** | **(0.22)** | **(0.20/0.18)** | **(0.16)** | **(0.26)** | **(0.21)** | **(0.29/0.27)** |
|  | **Conditional R²** | **(0.25)** | **(0.31/0.31)** | **(0.33)** | **(0.35)** | **(0.41)** | **(0.37/0.36)** |

**Table S3.2** Coefficients estimates (Odds ratio +/- 85% CIs) of the best models from the contemporary host and vector-centred analysis (*Analysis IV*) for ectoparasites only. Informative parameters are presented in bold. conditional R^2^ = variation explained by full model including random effects; marginal R^2^ = variation explained by fixed effects only as.

| Stage | Variable | *Fleas* | *I. trianguliceps* | *I. ricinus* |
| --- | --- | --- | --- | --- |
| Current year | Local-scale host abundance |  |  | **0.80 (0.69-0.93)** |
|  | Landscape-scale host connectivity |  |  |  |
|  | Metapopulation-scale host abundance |  | **1.55 (1.27-1.89)** | **1.39 (1.17-1.66)** |
| Individual | Weight |  | 1.79 (1.52-2.11) |  |
|  | Weight² | 1.18 (1.06-1.32) | 0.81 (0.71-0.93) |  |
|  | Sex:Female | 0.05 (0.03-0.07) |  | 0.52 (0.40-0.68) |
|  | Sex:Male | 0.10 (0.06-0.19) |  | 0.82 (0.53-1.25) |
|  | Weight:Sex:Female | 0.89 (0.75-1.07) |  | 1.20 (1.07-1.35) |
|  | Weight:Sex:Male | 1.12 (0.75-1.67) |  | 1.58 (1.20-2.10) |
| Model performance | Marginal R² | (0.03) | (0.09) | (0.06) |
|  | Conditional R² | (0.32) | (0.39) | (0.33) |

**Table S3.3** Coefficients estimates (Odds ratio +/- 85% CIs) of the best models from the contemporary host and vector-centred analysis (*Analysis IV*) for microparasites only. Informative parameters are presented in bold. conditional R^2^ = variation explained by full model including random effects; marginal R^2^ = variation explained by fixed effects only as.

| Stage | Variable | Scale | *Bartonella* | *B. microti* | *Hepatozoon* |
| --- | --- | --- | --- | --- | --- |
| Current year | **Host** | Local-scale abundance |  |  |  |
|  |  | Landscape-scale connectivity | **0.66 (0.54-0.82)** |  |  |
|  |  | Metapopulation-scale abundance |  |  | **2.11 (1.39-3.23)** |
|  | **Fleas** | Local-scale abundance |  | / |  |
|  |  | Landscape-scale connectivity | **1.67 (1.37-2.03)** | / |  |
|  |  | Metapopulation-scale abundance |  | / | **0.52 (0.32-0.84)** |
|  | ***I. trianguliceps*** | Local-scale abundance | / | **1.30 (1.05-1.62)** |  |
|  |  | Landscape-scale connectivity | / | **2.30 (1.69-3.12)** |  |
|  |  | Metapopulation-scale abundance | / |  |  |
|  | ***I. ricinus*** | Local-scale abundance | / |  |  |
|  |  | Landscape-scale connectivity | / |  |  |
|  |  | Metapopulation-scale abundance | / |  |  |
| Individual | Weight |  | 1.00 (0.87-1.15) | 1.82 (1.52-2.17) | 0.44 (0.35-0.56) |
|  | Weight² |  | 0.57 (0.49-0.66) |  | 1.34 (1.09-1.65) |
| Model performance | Marginal R² |  | (0.11) | (0.24) | (0.23/0.17/0.17) |
|  | Conditional R² |  | (0.32) | (0.47) | (0.37/0.34/0.36) |

**References**

Hassall, R. M. (2020). *Understanding the Generation and Dynamics of Bartonella Diversity in Fragmented Host Populations*. University of Aberdeen .

Simpson, V. R., Panciera, R. J., Hargreaves, J., McGarry, J. W., Scholes, S. F. E., Bown, K. J., & Birtles, R. J. (2005). Myocarditis and myositis due to infection with Hepatozoon species in pine martens (Martes martes) in Scotland. *Veterinary Record*, *156*(14), 442–446. https://doi.org/10.1136/vr.156.14.442
