# Supplementary material for: Patterns and drivers of vector-borne microparasites in a classic metapopulation
Source: Parasitology. 2023 Jul 31;150(10):866–82. doi: 10.1017/S0031182023000677 (PMC10577662; doi:10.1017/S0031182023000677)
Supplement: Supplementary file 1 [file S0031182023000677sup.zip › S0031182023000677sup003.docx]

Table S.4.1 Table outlining the predictions and results from the analysis of scale-associated lagged infection patterns (Analysis III) . Results that lined up with predictions are marked with a * while results that are unexpected are marked with a °.

| **Scale** | **Prediction** | **Result** |  |
| --- | --- | --- | --- |
| **Analysis II & III: Spatio-temporal patterns in infection probability** | | | |
| **Local**  **Contemporary** | All parasites were expected to be positively correlated with the local abundance of infected individuals, reflecting local aggregation. | *All parasites were locally aggregated, with infection probability increasing with the contemporary local abundance of infected hosts. |  |
| **Landscape**  **Contemporary** | All rodent-associated parasites (fleas, *I. trianguliceps*, *Bartonella* and *Babesia microti*) are expected to be positively correlated with infection connectivity, reflecting landscape-scale aggregation, in line with water vole dispersal driving infection dynamics. | **I. trianguliceps* were aggregated at the landscape-scale.  ° Fleas, °*Bartonella* and °*Babesia microti* were not aggregated at this scale. |  |
| **Metapopulation**  **Contemporary** | If there are strong annual variations in infection probability, the host generalist, *I. ricinus* is expected to be better predicted by metapopulation-scale total abundance (i.e. aggregation scale not in line with water vole dispersal). | **I. ricinus*, °fleas and *Hepatozoon* were positively correlated with metapopulation-scale abundance of infected individuals. |  |
| **Local**  **Lagged** | Ticks and tick-borne infections are expected to be more strongly positively correlated with infection abundance in the previous year than fleas and flea-borne infections. | **I. trianguliceps* and **B. microti* were positively correlated with lagged local-scale abundance of infected hosts.  °*Bartonella* were negatively correlated with infection abundance in the previous year.  *No effect seen for fleas. |  |
| **Landscape**  **Lagged** | Ticks and tick-borne infections are expected to be more strongly positively correlated with infection connectivity in the previous year, than fleas and flea-borne infections. | **B. microti* was positively correlated with infection connectivity in the previous year.  ° No effect seen for *I. trianguliceps.*  No effect was seen for **Bartonella* or *fleas. |  |
| **Metapopulation**  **Lagged** | If there are strong temporal patterns, *I. ricinus* ticks are expected to be positively correlated with metapopulation-scale total abundance in the previous year. | °*Bartonella,* *Hepatozoon* and °*I. ricinus* were negatively correlated with the metapopulation-scale abundance of infected hosts in the previous year. |  |

Table S.4.2 Table outlining the predictions and results from the lagged host and vector-centred analysis (Analysis V). Results that lined up with predictions are marked with a * while results that are unexpected are marked with a °.

| **Scale** | **Prediction** | **Result** |
| --- | --- | --- |
| **Analysis IV& V: Contemporary and lagged drivers of infection probability** | | |
| **Local**  **Contemporary** | All rodent-associated parasites (fleas, *I. trianguliceps*, *Bartonella* and *Babesia microti*) are expected to be positively correlated with host and/or vector abundances. | ° *I. ricinus* was negatively correlated with local host abundance.  **B. microti* was positively correlated with local vector (*I. trianguliceps*) abundance.  °No effect of local vector (flea) abundance on *Bartonella.*  ° No effect of local host abundance on fleas, *I. trianguliceps*, *Bartonella* and *Babesia microti.* |
| **Landscape**  **Contemporary** | All rodent-associated parasites (fleas, *I. trianguliceps*, *Bartonella* and *Babesia microti*) are expected to be positively correlated with host and/or vector connectivity, in line with water vole dispersal driving infection dynamics. | **B. microti* was positively correlated with vector (*I. trianguliceps*) connectivity but °not host connectivity.  **Bartonella* were positively correlated with vector (flea) and host connectivity.  °No effect of host connectivity on fleas, *I. trianguliceps.* |
| **Metapopulation**  **Contemporary** | No associations expected (*I. ricinus* not expected to be associated with the abundance of one possible host, other parasites expected to be associated with landscape connectivity measures) | °*I. trianguliceps*, °*I. ricinus* and *Hepatozoon* infection probability was positively correlated with the metapopulation-scale abundance of hosts. |
| **Local**  **Lagged** | Ticks and tick-borne infection are expected to be more strongly positively correlated with host and/or vector abundance in the previous year, than fleas and flea-borne infections. | **B. microti* were positively correlated with lagged *I. trianguliceps* abundance, but °negatively correlated with *I. ricinus* abundance.  ° No effect of lagged host abundance on *I. trianguliceps*. |
| **Landscape**  **Lagged** | Ticks and tick-borne infection are expected to be more strongly positively correlated with host and/or vector connectivity in the previous year, than fleas and flea-borne infections. | °Fleas were negatively correlated with landscape-scale host connectivity.  **Bartonella* was positively correlated with lagged vector (flea) connectivity, but °no effect of lagged host connectivity.  ° No effects seen for *I. trianguliceps* or *B. microti*. |
| **Metapopulation**  **Lagged** | No associations expected (*I. ricinus* not expected to be associated with the abundance of one possible host, other parasites expected to be associated with landscape connectivity measures). | Hepatozoon was negatively correlated with high metapopulation-scale flea and *I. ricinus* abundance in the previous year. |
